# On Injection of the Bronchial Tubes and Tubercular Cavities of the Lungs

**Published:** 1855-04

**Authors:** 


ART. IV.—*On Injection of the Bronchial Tubes and Tubercular Cavities of the Lungs.* By HORACE GREEN, M. D., *New York.*

THIS paper, which now comes to us in the shape of a very neat pamphlet of twenty pages, originally appeared in the American Medical Monthly. The subject of which it treats is one of great importance in practical medicine, and having excited considerable discussion recently in several foreign medical societies, and called forth a good many savage articles in the domestic medical press, Dr. GREEN has thought it proper again to bring the question before the profession of this country.

Dr. GREEN has occupied a large share of public attention since the claim he laid a few years since to certain discoveries in the topical medication of the air-passages; and very various and conflicting have been the views of both the discoveries and their author entertained by physicians. There are those who have not scrupled to affirm that the professor of theory and practice in the New York Medical College is a counterfeit and his discoveries a sham. There are others again, and we are of this number, who believe Dr. GREEN to be honest, rather enthusiastic and disposed to ride his hobby a little too far, but not the less truthful, and that his discoveries have brought within reach of topical medication structures which were previously inaccessible, whereby diseases that have hitherto proved mortal can now, in many instances, be completely arrested.

In our opinion, Dr. GREEN has been very harshly used. In the first place, the honor of discovery was refused him. This after a time being pretty generally conceded, the feasibility of carrying the sponge-armed probang below the vocal chords was denied in the most positive terms. The names of illustrious foreigners, with ERICHSEN and TROUSSEAU at their head, were arrayed against it. Men who expressed themselves as believing the operation practicable were hooted at. To this day there are those who hold it to be anatomically impossible to insert the probang beyond the rima glottidis, and who seriously believe that Dr. GREEN has, all this time, been mistaking the œsophagus for the trachea and douching the mucous membrane of that tube instead of that lining the interior of the air-passages. There are now, however, in this country alone, more than five hundred different medical men, many of them eminent as physicians, surgeons, and some of them teachers, who honestly believe they have seen the sponge-probang carried beyond the chords through the trachea and into its bifurcations, and as many more who have no doubt as to the entire practicability of the operation. We ourselves desire to be ranked among the latter number. There are, however, certain objections to the pamphlet under notice which we feel called upon to express; at the same time we here take occasion to say we shall be very glad if Dr. GREEN is able to answer them to our satisfaction.

The character of the witnesses who were present at the catheterism of the trachea of the Rev. Mr. McANN, (page 8) and the possibility of the procedure, render all doubt as to this case out of the question. We regret that we cannot say as much of the case which follows it. To give full credit to this requires a larger faith than even we lay claim to. In order that the grounds of our skepticism may not be misunderstood we quote the case entire:—

"In order to test, still further, the truth of the operation, a small air-tight, elastic bag was tied over the upper extremity of the tube, and on introducing the instrument, six or eight inches into the trachea of a gentleman, this little bag was inflated and collapsed a dozen times, by the acts of inspiration and expiration on the part of the patient. In performing this last experiment, an incident occurred (which, had the tube been shorter, might have proved an accident,) that is an additional proof of the position of the instrument. The tube which, as I have stated, is thirteen inches long, was introduced its whole length, so that the upper extremity was flush with the lips of the patient, the elastic bag, which is three inches long, only remaining out of his mouth. After the patient had filled and emptied the sac several times, I let go, for a moment, my thumb and finger hold of the extremity of the tube. Just then the patient made a strong inspiration, when the whole instrument, sac and all, was drawn suddenly in, and for a moment disappeared out of sight. Thrusting my fingers immediaiely into the throat of the patient, I could barely reach, at the base of the tongue, the upper extremity of the bag, which I seized with my thumb and finger, and drew the whole out together."

Allowing now that none of the bag, which was itself three inches long, entered the larynx, still thirteen inches of the tube according to Dr. G's. statement, were carried into the trachea and bronchial tubes, or about nine and a half inches into the larynx, trachea and left bronchus, and three and a half inches into the bronchial ramifications. This itself is enough to stagger any but the most credulous, but when we add two other inches for the length of bag that must also have been drawn into the larynx, the thing seems to us anatomically impossible if any reliance is to be placed in the measurements of the air-passages as laid down in the books.

After having satisfied himself and all who witnessed his operations, that catheterism of the larynx and trachea could be performed without difficulty, Dr. Green thinking there was no reason why medicinal agents should not be introduced through the tube that he used into the lungs, or directly into the bronchi and their terminations, he "determined to test the effect of a solution of nitrate of silver, applied directly and freely to the bronchi in disease of their membrane; also in disease of the lungs, to inject, if possible, the same solution into tubercular excavations."

On the 13th of October, 1854, he attempted this experiment for the first time. On the same day he commenced, in like manner, the treatment of other cases, and from that time until the date of his pamphlet, he treated for a longer or shorter period, thirty-two patients laboring under tubercular or bronchial diseases, by the direct introduction into the lungs of a strong solution of the nitrate of silver injected through the elastic tube. The first patient, a lady from Connecticut, was the subject of a large cavity in the apex of the left lung. Dr. GREEN passed one of HUTCHING'S elastic tubes (which is thirteen inches long) into the left bronchial division. "Through this tube, with a small glass syringe, I injected one drachm of a solution of nitrate of silver, of the strength of forty grains to the ounce of water, into the lung. No cough whatever, or any sense of suffocation, was produced by this operation, nor did the patient observe in the least the ordinary bitter taste of the solution. A few minutes after the operation she stated that she "felt a warm sensation" in the upper portion of the left lung, but no pain, or any unpleasant feeling whatever, followed the operation. Mrs. A. did not return to have the operation repeated until the 17th, four days afterwards, when she stated that for twenty-four hours after the use of the injecting tube, her cough and expectoration were both greatly diminished, that she had breathed with more freedom than before; that these favorable symptoms had continued, though not as marked as at first, up to the present time. She was therefore much disposed to have the operation repeated. The tube was again introduced through the trachea its entire length, and at this time one and a half fluid drachms of the solution were thrown into the lungs. The immediate results were the same as at first, but after some minutes, she began to cough, and expectorated easily, and at once, nearly two ounces of purulent matter, changed in its color and consistence, apparently, by its immediate contact with the argentine solution. Indeed, the expectorated matter presented precisely the appearance which is observed to take place with the purulent matter of an external ulcer when cauterized with the nitrate of silver. This changed condition of the expectoration was observed by several physicians who were present when the operation was performed. The relief which followed this last operation in Mrs. A's. case was still more marked and decided than in the first instance. Her cough she stated was much relieved, the expectoration yet more diminished, and her breathing was easier. A pain in the chest of which she had complained was removed; and during the two nights which followed the operation her sleep was better than it had been for a long period before. Mrs. A. remained until the 26th, during which time the elastic tube was introduced into the left bronchial division seven times, and on each occasion from one and a half to two drachms of a strong solution of the nitrate of silver were injected into the lungs. Her improvement was constant. She grew stronger, and gained flesh in this period; but, being obliged at this time to return to her home, she left with the intention of coming back to renew the treatment, in a few weeks."

Concerning this very remarkable case we will ask a single question, and with it close our notice of the pamphlet. By what law did this "argentine solution" ascend from the root—where it was applied—to the apex of the lung; and how did it excite a sensation of warmth in a cavity which was surely destitute of all sensibility?

